# Healthcare Providers’ Perspectives on the Involvement of Mental Health Providers in Chronic Pain Management

**DOI:** 10.3390/healthcare13202604

**Published:** 2025-10-16

**Authors:** Aziza Ali Alenezi, Amin K. Makhdoom, Rehab Abdullah Alanazi, Fahad Saad Z. Alanazi, Yusef Muhana Alenezi, Zaid Alkhalfi Alanazi, Naglaa A. Bayomy, Manal S. Fawzy

**Affiliations:** 1 University Health Center, Northern Border University, Arar 91431, Saudi Arabia; azizaali7890@gmail.com; 2 Department of Otolaryngology, King Abdulaziz Hospital, Ministry of National Guard, Alahsa 31982, Saudi Arabia; akm.nbu@gmail.com; 3 Family and Community Department, Faculty of Medicine, Northern Border University, Arar 91431, Saudi Arabia; dr.rehaby55@gmail.com (R.A.A.); fahdsz411@gmail.com (F.S.Z.A.); dr.yusefmd@gmail.com (Y.M.A.); zaidenazi@gmail.com (Z.A.A.); 4 Department of Anatomy, Faculty of Medicine, Northern Border University, Arar 91431, Saudi Arabia; najlaa.bayoumy@nbu.edu.sa; 5 Center for Health Research, Northern Border University, Arar 73213, Saudi Arabia

**Keywords:** chronic non-malignant pain, mental health integration, healthcare providers’ perceptions, Theoretical Domains Framework, interdisciplinary collaboration, Saudi Arabia

## Abstract

Background/Objectives: Chronic non-malignant pain (CNMP) affects 46.4% of adults in Saudi Arabia and often requires interdisciplinary care, including mental health services. Despite this need, mental health integration remains limited. This study explored healthcare providers’ perceptions of integrating mental health services into CNMP management and identified barriers and facilitators to interdisciplinary collaboration. Methods: A cross-sectional survey was conducted among 114 healthcare providers across Saudi Arabia. Using the Theoretical Domains Framework (TDF), domains such as knowledge, skills, beliefs about capabilities and consequences, reinforcement, and social influences were assessed. Data were analyzed using descriptive statistics, correlation analyses, and multiple regression. Results: Positive perceptions of mental health integration were significantly associated with beliefs about capabilities (r = 0.31, *p* = 0.001) and beliefs about consequences (r = 0.40, *p* < 0.001), as well as skills (r = 0.30, *p* = 0.001) and reinforcement (r = 0.26, *p* = 0.005). Multiple regression confirmed beliefs about capabilities (B = 0.208, *p* = 0.001) and consequences (B = 0.237, *p* < 0.001) as independent predictors, explaining 31.9% of the variance in perceptions (R^2^ = 0.319, adjusted R^2^ = 0.285). Emotional responses, such as stress, were potential barriers but did not independently predict perceptions. Systemic challenges included limited referral pathways and insufficient mental health resources. Conclusion: Confidence in professional abilities and recognition of the benefits of collaboration are key drivers of positive perceptions toward mental health integration in CNMP care. Interventions that enhance provider confidence, emphasize interdisciplinary benefits, and strengthen organizational support may improve engagement with mental healthcare services in Saudi Arabia.

## 1. Introduction

Chronic non-malignant pain (CNMP) is a prevalent and often debilitating condition affecting between 11% and 40% of the population worldwide [1]. It is a leading cause of disability, with significant adverse effects on physical, social, and emotional functioning [2]. Notably, CNMP frequently co-occurs with mental health conditions such as anxiety and depression, complicating management and exacerbating individual suffering [3].

In Saudi Arabia, CNMP management incorporates both pharmacological and non-pharmacological approaches [4]. Historically, opioids have played a central role; however, rising concerns about their long-term safety and an increasing opioid crisis have prompted greater scrutiny. Research indicates that opioid use among patients with CNMP rose from 8.6% in 2006 to 14.7% in 2015, with a reported prevalence of opioid use disorder at 29.6% among these patients [5].

The strength of the patient–provider relationship plays a crucial role in shaping patient outcomes across various areas of healthcare. Positive, trusting relationships enhance patient understanding, adherence, and satisfaction with care, directly influencing the quality of CNMP management [6]. Yet, pain management care is often fragmented, characterized by poor inter-provider communication and inadequate coordination between physical and mental health services, resulting in suboptimal outcomes [7]. Collaborative Care Management (CCM) is a team-based approach meant to address these challenges by coordinating treatment among multiple healthcare professionals [8]. While CCM is well established in the management of mental health disorders such as depression and anxiety, its adoption for CNMP is limited, despite the urgent need for integrated care due to frequent comorbidities [9,10].

**Figure 1:**
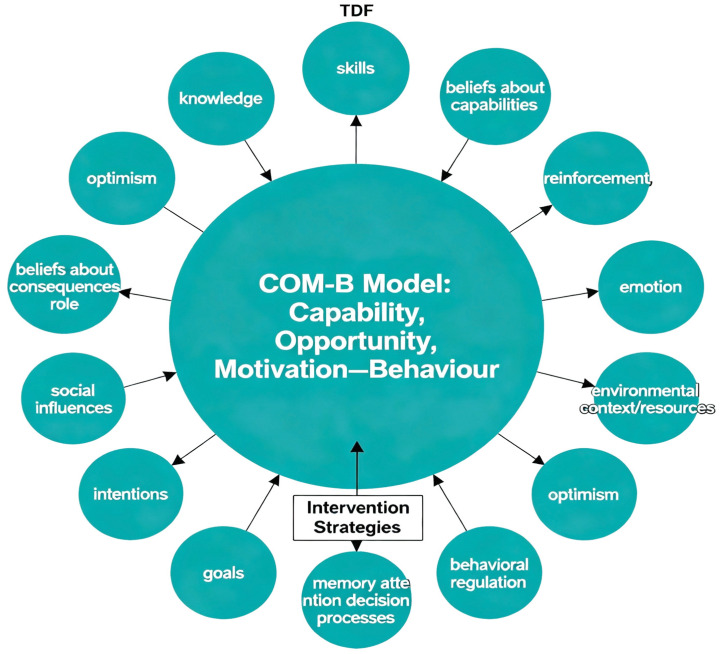
Summary of how the Theoretical Domains Framework (TDF) and its 14 behavioral domains map onto the COM-B model at the center of the Behaviour Change Wheel (BCW). TDF domains inform the determinants of capability, opportunity, and motivation, which collectively guide the development of targeted intervention strategies for provider behavior in chronic pain management.

To elucidate determinants of provider behavior in such complex contexts, the Theoretical Domains Framework (TDF) offers 14 domains derived from psychological theory, including knowledge, skills, beliefs about capabilities, and environmental context. The TDF is frequently integrated into the Behaviour Change Wheel (BCW), which systematically connects theory to practical intervention strategies via the Capability, Opportunity, Motivation—Behaviour (COM-B) model (Figure 1). This model posits that individuals must possess capability, opportunity, and motivation to enact a behavior [11]. Applying the TDF enables a thorough examination of healthcare providers’ beliefs and perceptions about interdisciplinary collaboration and helps clarify the behavioral factors shaping decision-making in CNMP management [11,12].

This study aims to explore healthcare providers’ perspectives on collaboration in CNMP management, with a particular focus on facilitators and barriers to integrating mental health services in Saudi Arabia. Framing the research within the TDF enables a systematic assessment of individual behaviors, attitudes, and contextual factors that influence provider engagement in collaborative care.

## 2. Materials and Methods

### 2.1. Study Design

This study employed a cross-sectional survey design to investigate healthcare providers’ perspectives on the involvement of mental healthcare providers (clinical psychologists and psychiatrists) in assessing and managing patients with chronic non-malignant pain (CNMP). A structured questionnaire, developed using the Theoretical Domains Framework (TDF), was used to explore these perspectives. The outcome variable was healthcare providers’ perception of interdisciplinary collaboration regarding the involvement of mental health professionals in CNMP management. The predictor variables were the TDF domains assessed in the questionnaire, including knowledge, skills, beliefs about capabilities, beliefs about consequences, reinforcement, social influences, environmental context and resources, intentions, and emotion [11,13].

Each predictor was measured through targeted survey items rated on a 5-point Likert scale (1 = strongly disagree to 5 = strongly agree). The survey was disseminated via email and professional networks, targeting healthcare providers involved in CNMP management.

### 2.2. Recruitment of Participants

The study population consisted of healthcare providers from public hospitals in regions such as Madinah, Arar, and Rafha within the Saudi public healthcare sector. Eligible participants were aged 18 or older and actively employed in the Saudi healthcare system, involved in CNMP management, including physicians, nurses, and other healthcare professionals. Recruitment was conducted through email invitations that included a cover letter, consent form, and survey link. Informed consent was obtained electronically to ensure confidentiality and adherence to ethical research guidelines. The sample size was calculated using Cohen’s Power Analysis for multiple regression with 11 independent variables [14]. A medium effect size (f^2^ = 0.15) with 80% power at a significance level of 0.05 indicated that a sample size of 110–138 participants would be sufficient for the reliable detection of meaningful relationships among variables.

### 2.3. Questionnaire Preparation

The TDF was employed to guide questionnaire development, informed by an extensive review of the literature and established behavior change models that synthesize 33 distinct psychological theories to address implementation challenges systematically [11]. The questionnaire consisted of two sections: demographics and theoretically framed questions based on the TDF domains. Demographic items included healthcare providers’ experience, education, expertise, current practice, pain management training (mandatory or voluntary), and self-assessed knowledge. Table 1 outlines our operationalization of each TDF domain as applied to collaborative management of chronic pain.

The initial draft comprised 74 items, covering the full range of standard TDF domains, based on validated guidelines from Huijg et al. [14]. Pilot testing was conducted with two primary care physicians, focusing on clarity and relevance. Consistent with published TDF research in pain and chronic disease settings, domains were excluded only when there was clear evidence of redundancy or limited applicability [15,16,17]. Specifically, pilot feedback revealed that certain domains (e.g., ‘Memory, Attention, and Decision Processes’) overlapped in content and intent with others, such as ‘Beliefs about Consequences,’ resulting in duplicative items. To strengthen questionnaire clarity while retaining content validity, we therefore limited inclusion to domains that were both relevant and non-redundant in this context, enhancing participant engagement and data reliability [16]. The final questionnaire comprised items addressing nine domains regarded as most pertinent to the study objectives and clinical context: knowledge, skills, beliefs about consequences, social influences, environmental context/resources, motivation/goals, behavioral regulation, beliefs about capabilities, and emotion. Each domain was assessed using two questions, each with a 5-point Likert scale (1 = strongly disagree to 5 = strongly agree). Demographic information (age, gender, profession, and years of experience) was also collected.

### 2.4. Data Collection Procedure

The questionnaire was distributed between January and June 2024 through research team acquaintances or sent via email links for online completion. All respondents provided informed consent before participating in the study, and the data were collected anonymously.

### 2.5. Ethical Statement

The study received approval from the Local Bioethics Committee at Northern Border University (approval no. 84/44/H, on 8 October 2023). Informed consent was obtained from all participants, and confidentiality was maintained throughout the study process. Data were securely stored on a password-protected computer accessible only to authorized research team members.

### 2.6. Data Management and Statistical Analysis

Data were entered into IBM SPSS Statistics v23 (IBM Corp., Armonk, NY, USA) with numerical coding for each questionnaire item. Participants were required to complete all items before submission. All items were positively framed, so that higher scores indicated positive outcomes or favorable attitudes regarding mental health provider involvement in CNMP management. Descriptive statistics summarized demographic characteristics and responses to TDF questionnaire items using means and standard deviations for continuous variables (e.g., age). At the same time, frequencies and percentages were reported for categorical variables (e.g., gender).

Pearson correlation coefficients were used to assess the relationships between independent variables representing TDF domains and the dependent variable (perception of interdisciplinary collaboration). Correlation coefficients were interpreted as follows: weak (0.1–0.3), moderate (0.3–0.5), and strong (0.5–1.0). Furthermore, an analysis of variance (ANOVA) was conducted to examine the relationship between healthcare providers’ perceptions and the predictor variables.

A multiple linear regression analysis was applied to identify significant predictors among independent variables: (1) knowledge, (2) skills, (3) beliefs about capabilities, (4) beliefs about consequences, (5) social influences, (6) environmental context and resources, (7) motivation, (8) goals, (9) behavioral regulation, (10) emotion, and (11) reinforcement concerning the dependent variable (perception of mental healthcare involvement in CNMP Management). R-squared values indicated the strength of these relationships with a significance level set at *p* < 0.05.

### 2.7. Data Screening and Assumption Checks

Before conducting the regression analysis, the data were screened to ensure that all assumptions of multiple linear regression were met. Normality of residuals was examined using histograms and Q-Q plots, linearity was assessed via scatterplots between predictors and the dependent variable, and homoscedasticity was evaluated through residual-versus-predicted plots. Multicollinearity was checked using the Variance Inflation Factor (VIF) values, with all predictors below the commonly accepted threshold of 5, indicating low collinearity. Outliers and influential cases were assessed using standardized residuals and Cook’s distance, and no observations required removal.

## 3. Results

### 3.1. Demographic Characteristics of the Study Participants

The study included 114 healthcare providers with diverse backgrounds (Table 2). The cohort’s mean age was 29.5 years (SD = 4.2, range: 23–48), with 59.6% reporting five years or less experience, indicating a predominantly early-career sample. Most participants worked in general hospitals (86.0%), with the majority from the Western Region (67.5%). Gender distribution was nearly even (52.6% female). Most held baccalaureate degrees (87.7%), and physicians comprised the largest professional group in the sample (46.5%), with details for other roles presented in Table 2.

### 3.2. Correlation Between Key Domains

Table 3 summarizes the key correlations between domains. Healthcare providers’ perceptions were significantly positively associated with reinforcement (r = 0.26, *p* = 0.005), beliefs about capabilities (r = 0.31, *p* = 0.001), beliefs about consequences (r = 0.40, *p* < 0.001), and skills (r = 0.30, *p* = 0.001). Notably, perceptions were also significantly negatively correlated with emotions (r = –0.29, *p* = 0.002), indicating that stronger negative emotional responses were linked to less favorable attitudes toward integrating mental healthcare in chronic pain management.

Other key associations included reinforcement with motivation and goals (r = 0.51) and with emotions (r = 0.71). Additionally, significant correlations were found between motivation and intentions (r = 0.61) and between skills and emotions (r = 0.59). Reporting focused on statistically significant relationships most relevant to understanding provider attitudes in the context of TDF domains.

Overall, these findings suggest that positive perceptions of mental health involvement are facilitated by reinforcement, skills, and constructive beliefs, while heightened negative emotional responses may act as a barrier, underscoring the importance of the emotion’s domain in shaping provider perspectives.

### 3.3. Multiple Regression Analysis

A multiple linear regression was conducted to examine the extent to which the TDF domains predicted healthcare providers’ perceptions of mental healthcare involvement in CNMP management. The regression model was statistically significant, F(10, 102) = 4.77, *p* < 0.001 (Table 4), explaining 31.9% of the variance in perceptions (R^2^ = 0.319, adjusted R^2^ = 0.285). This indicates that the set of predictors collectively contributes significantly to explaining the dependent variable.

### 3.4. Regression Results

Table 5 shows the unstandardized coefficients of the predictors. Beliefs about capabilities (B = 0.208, *p* = 0.001) and beliefs about consequences (B = 0.237, *p* < 0.001) emerged as significant positive predictors of perceptions regarding mental healthcare involvement. Skills showed a positive but non-significant association (*p* = 0.087). Other variables, including reinforcement, knowledge, intentions, social influence, environmental context/resources, and emotions, were not significant predictors.

These findings suggest that healthcare providers’ confidence in their abilities and recognition of the impact of mental healthcare are key factors in fostering positive perceptions. In contrast, other domains, including emotional responses, did not independently predict perceptions in the regression model.

## 4. Discussion

The findings of this study provide clear evidence regarding the factors that shape healthcare providers’ perspectives on the role of mental health professionals in managing chronic pain. While demographic features such as age, gender, education, and professional background did not significantly influence pain management knowledge or skills, providers’ beliefs about their own capabilities and beliefs about the consequences of mental healthcare involvement emerged as the most important predictors. These results align with our objective of identifying the determinants of provider perceptions, highlighting that attitudinal and cognitive factors, rather than demographic characteristics, drive receptivity to interdisciplinary models of pain management.

This pattern is echoed in international research and established theoretical models. For example, the TDF and the Theory of Planned Behavior highlight the central role of self-efficacy and outcome expectations in behavior change [18,19]. Similar findings have been observed in studies examining the implementation of collaborative pain management in various healthcare settings, where provider confidence and positive expectations regarding interdisciplinary involvement are crucial for successful adoption [7,20,21].

Consistent with our data, providers who felt confident in their capabilities were more likely to perceive mental healthcare involvement as beneficial [21,22]. The belief in positive consequences was a strong predictor of favorable attitudes, supporting existing research on the impact of perceived outcomes in healthcare decision-making [22,23,24]. Wong et al. also demonstrate that providers’ beliefs about the benefits of integrating mental health services significantly influence engagement in collaborative care [25].

Previous literature has demonstrated that interventions enhancing provider self-efficacy and clarifying expected patient outcomes lead to increased adoption of non-pharmacological pain management approaches, underscoring our findings [26,27,28,29,30]. These initiatives have also shown that training in psychological skills and interprofessional communication further supports the successful integration of mental health into pain care [29,31,32].

While skills, intentions, and reinforcement appeared as secondary predictors in this study, their crucial roles as indirect or foundational contributors are well documented. Eccleston et al. emphasize that pain assessment and psychological support skills are essential to interdisciplinary care [31]. Kroenke and Cheville discuss provider intentions as central to adopting holistic, patient-centered care models in place of opioid mono-therapy [33]. Fisher et al. confirm the crucial influence of psychological skills and intentional collaboration in managing recurrent pain [34]. This is supported by systematic reviews that show skills and teamwork form the backbone of successful collaborative models [35,36].

The present analysis identified beliefs about capabilities and beliefs about consequences as key facilitators of positive perceptions regarding the integration of mental health services in CNMP management. Healthcare providers who feel confident in their professional abilities and recognize the benefits of collaborating with mental health professionals are more likely to engage in interdisciplinary care, which can ultimately lead to improved patient outcomes. In contrast, emotional responses, such as stress or anxiety, may act as barriers, potentially reducing favorable perceptions; however, these were not significant independent predictors in the regression analysis. Other factors, including reinforcement, social influence, and environmental context, did not independently predict perceptions; however, they may play a supportive role in fostering collaboration. Overall, these findings suggest that interventions focusing on enhancing provider confidence, emphasizing the benefits of collaboration, and providing supportive organizational resources may be particularly effective in promoting engagement with mental healthcare services.

Our findings on environmental context and resources point to challenges such as limited specialist availability and fragmented referral pathways. Yet these are not unique to Saudi Arabia; similar barriers have been reported internationally [37]. Qureshi and colleagues further note that although mental health integration in primary care is advancing in Saudi Arabia, a standardized referral pathway is lacking, affecting both primary and specialized care [38].

Evidence mapping in Saudi Arabia reveals ongoing gaps in patient-centric management and sparse data on chronic pain prevalence and outcomes, impeding informed policy and service planning [39]. In comparison, the international literature highlights similar gaps in patient-centered research, with an increasing focus on outcome-based approaches [40]. Saudi policy makers should therefore prioritize targeted resource allocation and patient-centered care pathways to address local needs [39].

Chronic pain is a complex condition influenced by biological, psychological, and social factors, yet management often remains dominated by a biomedical perspective. [41]. This narrow focus can lead to essential aspects being overlooked, such as the psychological needs of patients and the role of effective communication [42]. Many patients report experiences of invalidation, dismissal, or the belief that their pain is psychogenic rather than real, which can worsen physical and mental health outcomes and act as barriers to appropriate care [42,43]. In Saudi Arabia, these challenges are compounded by deficient referral systems and inadequate training in the psychiatric aspects of pain management. A semi-systematic review highlighted the lack of reliable prevalence data and limited patient-centric outcomes in the region, underlining the need for more integrated approaches (26). Furthermore, formal training in pain management is limited: Wazqar reported that only a small proportion of nurses had received post-licensing education in pain [44]. At the same time, Al-Samarkandi found that fewer than half of Saudi nursing students had received advanced pain training, with even those who had received training showing limited proficiency in non-pharmacological methods [45]. These findings emphasize the importance of training and education to support the integration of mental health within pain management.

The present study’s results suggest that interventions specifically designed to enhance provider confidence (beliefs about capabilities) and reinforce providers’ expectations of positive patient outcomes (beliefs about consequences) are likely to be particularly effective in advancing integrated care. Evidence from international CCM interventions shows that skills-building workshops, multidisciplinary feedback sessions, and ongoing peer mentorship can foster such beliefs and significantly improve provider engagement with collaborative mental health integration [46,47,48,49,50,51]. Embedding such approaches into professional development for Saudi clinicians may directly address the barriers highlighted by the current findings, providing a practical pathway for improving policy and practice.

### 4.1. Implications and Recommendations

The findings highlight the need for policy initiatives in Saudi Arabia that formally integrate mental health into chronic pain management. A nationwide Collaborative Care Model (CCM) could support this by fostering team-based care with clear roles for each provider, including mental health professionals. Training programs grounded in the Theoretical Domains Framework (TDF) should target key areas such as building provider confidence (capability) and raising awareness of the benefits of collaboration (consequences). Embedding these skills into continuing professional development and institutional policies would help ensure that psychologists, social workers, and primary care teams work in coordination. Such policy-driven interventions could reduce fragmentation of care and promote a more holistic, patient-centered approach to managing chronic pain.

To successfully implement a nationwide collaborative care model (CCM) for chronic pain in Saudi Arabia, it is essential to foster both attitudinal and skills-based readiness among providers. Training programs built on the TDF should focus on developing confidence, outlining expected outcomes, and promoting interdisciplinary communication. Policy initiatives must also prioritize structured referral pathways and adequate resourcing—key lessons from successful international models. Active involvement of mental health professionals alongside primary care teams will promote holistic, patient-centered pain management.

### 4.2. Strengths and Limitations

This study focused on a predominantly younger, Saudi-trained cohort, aligning with Vision 2030’s objectives for the workforce. However, this may limit the generalizability to older or international provider populations. The sample size approached statistical recommendations; however, reliance on self-report may have introduced bias, and the cross-sectional design precludes causality.

A critical appraisal of our use of the TDF indicates that, while the instrument was based on validated sources and underwent pilot testing for clarity and overlap, some domains may not have been fully or distinctly captured. The potential for conceptual overlap, particularly between domains such as beliefs about capabilities, goals, and intentions, raises the possibility that some secondary predictors may not have been significant due to collinearity. In future research, employing more concise models and undertaking comprehensive psychometric evaluation would serve to clarify the unique contributions of each domain and optimize instrument validity.

By applying the TDF in an underexplored context, our study provides new insight into barriers and facilitators for integrating mental healthcare in chronic pain management within Saudi Arabia and globally.

## 5. Conclusions

In conclusion, this study highlights the pivotal influence of healthcare providers’ beliefs about their capabilities and the consequences of integrating mental healthcare into CNMP management. The findings suggest that Saudi healthcare institutions could enhance CNMP management by implementing policies that support systematic reinforcement, ongoing pain education, and collaborative training.

## Figures and Tables

**Table 1:** The application of the Theoretical Domains Framework (TDF) in assessing healthcare providers’ perspectives on collaborative CNMP management.

Domain	Definition	Constructs	Application in This Study
Knowledge	Awareness of the existence of something.	- Knowledge of condition/scientific rationale - Procedural knowledge - Knowledge of the task environment	Healthcare providers’ understanding of CNMP, its psychological aspects, and the role of mental health professionals in its management.
Skills	Ability or proficiency acquired through practice.	- Skills development - Competence - Ability - Interpersonal skills - Practice - Skill assessment	Providers’ proficiency in collaborating with mental health professionals and implementing integrated care strategies for CNMP patients.
Social/Professional Role and Identity	A coherent set of behaviors and displayed personal qualities of an individual in a social or work setting.	- Professional identity - Professional role - Social identity - Professional boundaries - Professional confidence - Group identity - Leadership - Organizational commitment	Providers’ perception of their role in CNMP management and their identity as collaborators with mental health professionals.
Beliefs about Capabilities	Acceptance of the truth, reality, or validity about an ability, talent, or facility that a person can put to constructive use.	- Self-confidence - Perceived competence - Self-efficacy - Perceived behavioral control - Empowerment - Professional confidence	Providers’ confidence in their ability to effectively collaborate with mental health professionals in CNMP management.
Beliefs about Consequences	Acceptance of the truth, reality, or validity of outcomes of a behavior in a given situation.	- Outcome expectancies - Anticipated regret - Consequents	Providers’ beliefs regarding the outcomes of collaborating with mental health professionals, such as improved patient outcomes or workflow efficiency.
Reinforcement	Increasing the probability of a response by arranging a dependent relationship, or contingency, between the response and a given stimulus.	- Rewards - Incentives - Punishment - Consequents - Contingencies - Sanctions	External factors that encourage or discourage providers from engaging in collaborative practices, such as institutional rewards or penalties.
Intentions	A conscious decision to perform a behavior or a resolve to act in a certain way.	- Stability of intentions - Stages of change model	Providers’ commitment and readiness to engage in collaborative care with mental health professionals.
Goals	Mental representations of outcomes or end states that an individual wants to achieve.	- Goal priority - Goal/target setting - Action planning - Implementation intention	Specific objectives providers aim to achieve through collaboration, such as enhanced patient care or professional development.
Memory, Attention, and Decision Processes	The ability to retain information, focus selectively on aspects of the environment, and choose between two or more alternatives.	- Memory - Attention control - Decision making - Cognitive overload/tiredness	Cognitive factors affecting providers’ ability to engage in and sustain collaborative practices.
Environmental Context and Resources	Any circumstance of a person’s situation or environment that discourages or encourages the development of skills and abilities, independence, social competence, and adaptive behavior.	- Environmental stressors - Resources/material resources - Organizational culture/climate - Barriers and facilitators	Organizational and systemic factors that influence collaboration include the availability of mental health professionals, institutional policies, and the allocation of resources.
Social Influences	Those interpersonal processes that can cause individuals to change their thoughts, feelings, or behaviors.	- Social pressure - Social norms - Group conformity - Social support - Power - Intergroup conflict - Group identity - Modeling	Influence of colleagues, supervisors, and the broader medical community on providers’ attitudes and behaviors toward collaboration.
Emotion	A complex reaction pattern, involving experiential, behavioral, and physiological elements, by which the individual attempts to deal with a personally significant matter or event.	- Fear - Anxiety - Stress - Depression - Positive/negative affect - Burn-out	Emotional responses that may affect providers’ willingness or ability to collaborate, such as stress, anxiety, or job satisfaction.
Behavioral Regulation	Anything aimed at managing or changing objectively observed or measured actions.	- Self-monitoring - Breaking a habit - Action planning	Strategies providers use to initiate and maintain collaborative behaviors, including self-monitoring and planning.
Optimism	The confidence that things will happen for the best or that desired goals will be attained.	- Optimism - Pessimism - Unrealistic optimism	Providers’ outlook on the potential success and benefits of integrating mental health services into CNMP management.

**Table 2:** Demographic and professional characteristics of study participants (*n* = 114).

Variable	* Category	*n* (%)
Sex	Female Male	60 (52.6%) 54 (47.4%)
Years of Experience	<1 year 1–3 years 3–5 years 5–10 years 10–15 years 15–20 years >20 years	29 (25.4%) 25 (21.9%) 14 (12.3%) 16 (14.0%) 19 (16.7%) 4 (3.5%) 7 (6.1%)
Hospital Type	General Specialty Primary Care	98 (86.0%) 13 (11.4%) 3 (2.6%)
Region	Western Northern Border Riyadh	77 (67.5%) 35 (30.7%) 2 (1.8%)
Education	Baccalaureate Master’s Doctorate	100 (87.7%) 6 (5.3%) 8 (7.0%)
Profession	Medicine Nursing Pharmacy Physical Therapy Others	53 (46.5%) 34 (29.8%) 11 (9.6%) 9 (7.9%) 7 (6.1%

* Categories are mutually exclusive.

**Table 3:** Correlation Matrix (Domains).

		Perception	Reinforcement	Motivation and Goals	Belief About Capabilities	Belief About Consequences	Skills	Knowledge	Intentions	Social Influence	Environmental Context/Resources	Emotions
Perception	Person correlation	1	0.264 **	0.071	0.309 **	0.396 **	0.298 **	0.099	0.046	−0.123	0.154	0.293 **
	Sig. (2-tailed)		0.005	0.458	0.001	0.000	0.001	0.299	0.626	0.194	0.103	0.002
Reinforcement	Person correlation	0.264 **	1	0.509 **	0.233 **	0.387 **	0.459 **	0.289 **	0.374 **	−0.163	0.292 **	0.713 **
	Sig. (2-tailed)	0.005		0.000	0.017	0.000	0.000	0.002	0.000	0.085	0.002	0.000
Motivation and goals	Person correlation	0.071	0.509 **	1	0.148	0.197 *	0.246 **	0.350 **	0.606 **	0.054	0.172	0.533 **
	Sig. (2-tailed)	0.458	0.000		0.118	0.037	0.009	0.000	0.000	0.570	0.068	0.000
Belief about capabilities	Person correlation	0.309 **	0.223 *	0.148	1	0.083	0.210	0.426 **	0.185 *	0.142	0.267 **	0.232 *
	Sig. (2-tailed)	0.001	0.017	0.118		0.380	0.025	0.000	0.050	0.134	0.004	0.013
Belief about consequences	Person correlation	0.396 **	0.387 **	0.197 *	0.083	1	0.189 *	0.291 **	0.217 *	−0.059	0.055	0.362 **
	Sig. (2-tailed)	0.000	0.000	0.037	0.380		0.045	0.002	0.021	0.538	0.564	0.000
Skills	Person correlation	0.298 **	0.459 **	0.246 **	0.210 *	0.189 *	1	0.180	0.382 **	−0.053	0.617 **	0.592 **
	Sig. (2-tailed)	0.001	0.000	0.009	0.025	0.045		0.057	0.000	0.579	0.000	0.000
Knowledge	Person correlation	0.099	0.289 **	0.350 **	0.426	0.291 **	0.180	1	0.251 **	0.251 **	0.114	0.331 **
	Sig. (2-tailed)	0.299	0.002	0.000	0.000	0.002	0.057		0.007	0.007	0.229	0.000
Intentions	Person correlation	0.046	0.374 **	0.606 **	0.185 *	0.217 *	0.382 **	0.251 **	1	0.151	0.327 **	0.98 **
	Sig. (2-tailed)	0.626	0.000	0.000	0.050	0.021	0.000	0.007		0.109	0.000	0.000
Social Influence	Person correlation	−0.123	−0.163	0.054	0.142	−0.059	−0.053	0.251 **	0.151	1	0.175	−0.143
	Sig. (2-tailed)	0.194	0.085	0.570	0.134	0.538	0.579	0.007	0.109		0.064	0.131
Environmental Context/Resources	Person correlation	0.154	0.292 **	0.172	0.267 **	0.055	0.617 **	0.114	0.317 **	0.175	1	0.317 **
	Sig. (2-tailed)	0.103	0.002	0.068	0.004	0.564	0.000	0.229	0.000	0.0674		0.001
Emotions	Person correlation	−0.293 **	0.713 **	0.533 **	0.232 *	0.362 **	0.592 **	0.331 **	0.498 **	−0.143	0.317 **	1
	Sig. (2-tailed)	0.002	0.000	0.000	0.013	0.000	0.000	0.000	0.000	0.131	0.001	

** Correlation is significant at the 0.01 level (2-tailed). * Correlation is significant at the 0.05 level (2-tailed).

**Table 4:** The table presents the results of the ANOVA for the regression model, which tests whether the predictors together explain a significant proportion of variance in the outcome.

	Model	Sum of Squares	df	Mean Square	F	Sig
1	Regression	14.270	10	1.427	4.771	0.000
	Residual	30.508	102	0.299		
	Total	44.779	112			

The dependent variable is the perception of mental healthcare involvement in CNMP management. Predictors include emotion, social influences, beliefs about consequences, environmental context/resources, motivation and goals, knowledge, intention, skills, and reinforcement.

**Table 5:** Regression analysis.

Model	Unstandardized Coefficients B	St. Error	Unstandardized Coefficients Beta	t	Sig.
Constant	2.106	0.448		4.706	0.000
Reinforcement	−0.019	0.088	−0.027	−0.213	0.832
Motivation and goals	0.017	0.076	0.027	0.227	0.821
Belief about capabilities	0.208	0.059	0.329	3.499	0.001
Belief about consequences	0.237	0.057	0.382	4.129	0.000
skills	0.220	0.128	0.216	1.726	0.087
Knowledge	−0.090	0.056	−0.165	−1.599	0.113
Intentions	−0.109	0.070	−0.176	−1.564	0.121
Social influence	−0.044	0.075	−0.054	−0.582	0.562
Environmental context/resources	−0.024	0.090	−0.030	−0.270	0.788
Emotions	0.089	0.126	0.100	0.706	0.482

The dependent variable is the perception of mental healthcare involvement in CNMP Management. St: standard, Sig.: significance (at the level 0.05).

## Data Availability

The original contributions presented in this study are included in the article. Further inquiries can be directed to the corresponding author.

## Abbreviations

The following abbreviations are used in this manuscript:

BCW	Behaviour Change Wheel
CNMP	Chronic non-malignant pain
CCM	Collaborative Care Management
COM-B	Capability, Opportunity, Motivation-Behaviour
TDF	Theoretical Domains Framework
